# On the evolution of protein–adenine binding

**DOI:** 10.1073/pnas.1911349117

**Published:** 2020-02-20

**Authors:** Aya Narunsky, Amit Kessel, Ron Solan, Vikram Alva, Rachel Kolodny, Nir Ben-Tal

**Affiliations:** ^a^Department of Biochemistry and Molecular Biology, George S. Wise Faculty of Life Sciences, Tel Aviv University, 69978 Ramat Aviv, Israel;; ^b^Department of Protein Evolution, Max Planck Institute for Developmental Biology, 72076 Tübingen, Germany;; ^c^Department of Computer Science, University of Haifa, Mount Carmel, 3498838 Haifa, Israel

**Keywords:** molecular evolution, molecular recognition, computational biology, ligand binding, structural biology

## Abstract

Proteins’ interactions with ancient ligands may reveal how molecular recognition emerged and evolved. We explore how proteins recognize adenine: a planar rigid fragment found in the most common and ancient ligands. We have developed a computational pipeline that extracts protein–adenine complexes from the Protein Data Bank, structurally superimposes their adenine fragments, and detects the hydrogen bonds mediating the interaction. Our analysis extends the known motifs of protein–adenine interactions in the Watson–Crick edge of adenine and shows that all of adenine’s edges may contribute to molecular recognition. We further show that, on the proteins' side, binding is often mediated by specific amino acid segments (“themes”) that recur across different proteins, such that different proteins use the same themes when binding the same adenine-containing ligands. We identify numerous proteins that feature these themes and are thus likely to bind adenine-containing ligands. Our analysis suggests that adenine binding has emerged multiple times in evolution.

## Significance

How proteins evolved to recognize and bind their ligands is a key mystery in protein function evolution. To explore this mystery, we study how proteins bind adenine, an ancient fragment. We characterize physicochemical patterns of protein–adenine interactions and link these to proteins’ evolutionary origins. In conflict with previous findings, we see that all of adenine’s hydrogen donors and acceptors have been used to bind proteins, and that adenine binding is likely to have emerged multiple times in evolution. To identify adenine-binding sites of shared origin, we use “themes”: short amino acid segments suggested to constitute evolutionary building blocks. We detect specific themes that are engaged in adenine binding; the detection of these in a protein’s sequence might reveal its function.

Protein function is the driving force of protein evolution (1, 2), and one of the most common and essential functions is ligand binding (3). Accordingly, understanding the processes by which proteins evolved to recognize and bind their ligands is a fundamental aim in the investigation of protein evolution. One potential approach to deriving such an understanding is to focus on ancient ligands—or on specific fragments of such ligands—that interact with numerous different proteins. In particular, for a given ligand (or ligand fragment), it could be useful to analyze how different proteins of common or disparate evolutionary origin interact with the ligand and to use that analysis to gain insights about how such interactions developed over the course of evolution.

Nucleotide cofactors are one class of ligands common in extant organisms, and, of these, nucleotides that serve as enzyme cofactors in key metabolic reactions are particularly prevalent. The biological importance and pervasiveness of nucleotides suggest that nucleotide binding is among the oldest and most conserved protein–ligand interactions. This idea is consistent with the suggested dominance of catalytic RNA molecules on the primordial Earth (4, 5). Adenine is a fragment of most nucleotide cofactors, and many protein–nucleotide interactions involve interactions between the protein and adenine. These interactions occur not merely because adenine is a fragment of nucleotides (indeed, it is a fragment of many other ligands) but also because of its chemical nature; that is, adenine includes several groups that participate in a variety of interactions used for binding: electrostatic and hydrogen bonding as well as aromatic and other nonpolar interactions (3). Although adenine is not the active component of protein–nucleotide binding, it serves as a “molecular handle” that increases binding affinity (6, 7) and specificity (8). Taken together, these features suggest that adenine is a potentially promising candidate for analyzing the evolutionary development of molecular recognition in proteins.

Accordingly, herein, we study the evolution of protein–adenine binding. Our investigation comprises two main elements: First, we characterize physicochemical adenine-binding patterns across a large set of adenine-binding proteins, extending previous studies of adenine-binding or adenine-cofactor-binding motifs (9–11) to provide a more comprehensive catalog of such patterns. Second, we analyze our set of proteins to identify groups with common evolutionary origin. In general, to deduce the common evolutionary origin of protein parts, scholars typically rely on sequence similarity (12, 13) and specifically seek to identify proteins with common domains. Here, we identify common evolutionary origins on the basis of “themes”: i.e., recurring protein segments at the subdomain level (13) (see below for further details). We then relate the two sets of data to draw conclusions about the evolution of adenine binding.

**Fig. 1.**
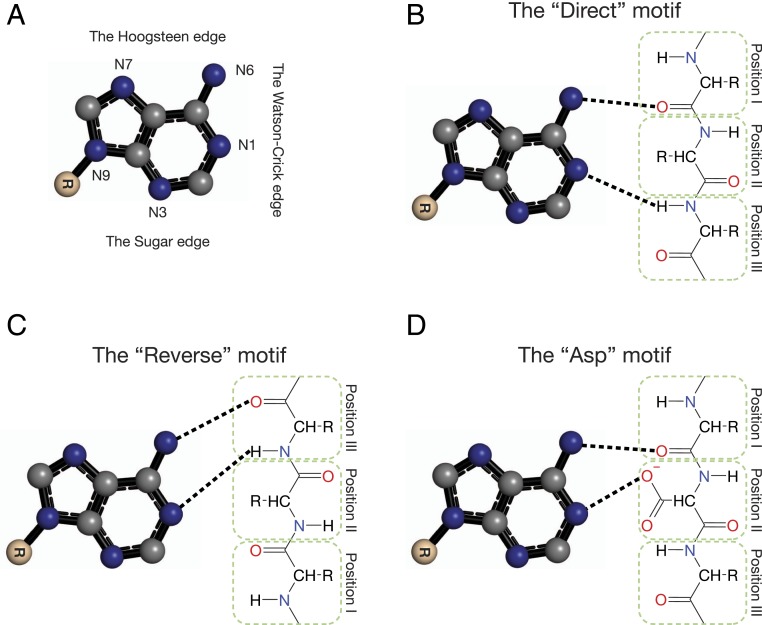
Previously documented adenine-binding motifs. Only the three most common ones are presented: (*A*) Adenine with the conventional atom numbering and binding edges. Carbon atoms are marked as gray spheres, nitrogen atoms in blue and oxygen atoms in red. (*B*, *C*, and *D*) The “direct,” “reverse,” and “Asp” motifs of adenine binding along the Watson–Crick edge. Hydrogen bonds are marked with dashed lines. Adapted from ref. 10, with permission from Elsevier.

Adenine is a planar, triangle-like molecule with three edges that can hydrogen-bond with its environment: the Watson–Crick edge, the Hoogsteen edge, and the sugar edge (Fig. 1*A*). Of these, only the former has been reported to bind proteins. Studies in the 1980s and 1990s were first to suggest an adenine-binding motif (14, 15); the motif includes carbonyl and amide groups within a protein loop that hydrogen-bond the N6 and N1 nitrogen atoms of the Watson–Crick edge of adenine. Denessiouk and coworkers extended the analysis to include proteins that bind adenosine 5′-triphosphate (ATP), nicotinamide adenine dinucleotide (NAD), flavin adenine dinucleotide (FAD), *S*-adenosyl methionine (SAM), and coenzyme A (CoA) (9–11). They suggested a general scheme for adenine-binding motifs: three amino acid positions located on a protein loop, which hydrogen-bond with adenine’s N1 and N6 groups, in three variants—the “direct motif,” the “reverse motif,” and the “Asp motif” (Fig. 1 *B*–*D*). In the first two variants, only the backbone interacts with adenine. This may suggest that these two variants are ancient adenine-binding modes and that the amino acid sequences are, in general, variable, and determined mostly to satisfy fold constraints. In the “Asp” motif variant, found mostly in the adenine-binding sites of NAD-binding proteins (Fig. 1*D*), the side chain of a negatively charged amino acid (mostly aspartate) hydrogen-bonds adenine’s N6. Subsequent studies validated these findings, emphasizing that adenine binding involves only adenine’s Watson–Crick edge (16–19), such that hydrogen-bonding opportunities in the Hoogsteen and sugar edges are not exploited. The latter observation implies that all recorded adenine-binding events diverged from a single evolutionary event—although the various studies do not explicitly state this conclusion.

Herein, we expand on these studies by analyzing protein–adenine interactions in a nonredundant representative set of 985 protein–adenine Protein Data Bank (PDB) complexes. We study the binding patterns from the perspectives of both the adenine and the binding proteins, using a computational pipeline (Common Binding [“ComBind”]) that we developed for this purpose. When considering the perspective of the adenine, we superimpose the adenine fragments, taking advantage of their rigid and planar shapes, and analyze the patterns of hydrogen bonding, in line with other studies (e.g., refs. 20–23); hydrogen bonds are prevalent in ligand-binding sites and are important for the specificity of protein–ligand interactions (24–33); see *SI Appendix* for further discussion of the choice to focus on hydrogen bonds. In conflict with the studies noted above, we find that all, rather than only some, hydrogen bond donors and acceptors in adenine can participate in the binding, which is often mediated by water molecules.

To trace the evolutionary process by which protein families, superfamilies, and folds emerged and continue to evolve, scholars search for links between their sequences, structures, and functions (5, 34–42). As noted above, the current view holds that domains are the principal evolutionary units and that the diversity of proteins emerged from the combinatorial shuffling of domains (40, 43–46). But how did the domains emerge? Several researchers have suggested that domains emerged from combinations of shorter peptides, originating in the RNA world (4, 5, 12, 47–49); presumably, peptides that could carry out molecular functions were favored and survived. In support of this mechanism, scholars have demonstrated that peptides with fewer than 55 amino acids can bind small ligands and even catalyze chemical reactions (50–52). The peptides were designed to include recurring sequence- and structure-similar motifs (like the Walker motifs in P-loops) (53). In further support, Keefe and Szostak showed that, starting from a library of 80-residue random peptides and using directed evolution, they can identify peptides that bind ATP, and their fraction increased with each in vitro selection round (54). A follow-up structural study revealed that, among various ATP-binding proteins generated randomly in the laboratory by this in vitro procedure, the one with the highest affinity to ATP had a novel fold and yet bound adenine using hydrogen bonds and aromatic stacking interactions in a way that resembled known protein–adenine binding (55).

The notion that domains evolved by combination of shorter peptides is also supported by computational studies aiming to detect motifs which are common features of proteins with similar function (49, 56–62). Some focused on proteins–nucleotides interactions: the Nucleotide Binding Database used “elementary functional loops” to identify recurring motifs in the binding of nucleotide cofactors (63), and Gherardini et al. (64) identified nucleotide-binding motifs in the Hoogsteen, sugar, and Watson–Crick edges. Recently, Nepomnyachiy et al. (13) introduced an algorithm to detect “themes”—sequences of 35 to 200 amino acids that recur in protein space. The authors’ focus on themes was motivated by the idea that the recurrence of a given segment across different protein chains is likely to indicate that the segment participates in an important biological function. The themes they detected formed complex patterns, consistent with evolution by duplication and divergence. These patterns suggest that themes, rather than domains, may be evolutionary building blocks.

Herein, building on the notion that themes may be evolutionary building blocks, we analyze our dataset to identify proteins that share themes in which at least one residue is involved in adenine binding. We then relate our findings to our physicochemical data. This approach enables us to explore the possibility that protein–adenine interactions are facilitated by certain themes and to attempt to delineate the structural and evolutionary relationship between these themes in different adenine-binding sites. We find that some themes bind only specific cofactors whereas others are shared by different cofactors. Moreover, we find that proteins sharing the same themes in their adenine-interaction sites tend to bind adenine via the same interaction patterns. This could guide experimental effort to reveal the origin of molecular recognition. More broadly, our analysis supports the putative role of themes as evolutionary building blocks of modern proteins and provides a link between themes and the emergence of a specific biological function at the atomic level.

## Results

### Analyzing Protein–Ligand Interactions Using ComBind.

**Fig. 2.**
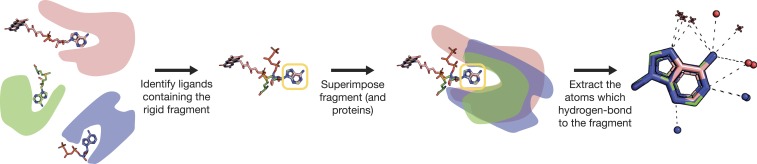
The ComBind methodology for detecting protein–fragment interactions among multiple and highly dissimilar proteins. The cofactors are represented as sticks, with the carbons of each cofactor colored differently than those of the other cofactors, where nitrogen, oxygen, and phosphorus atoms are colored by type. The rigid fragment (in this example, adenine) is enclosed by the yellow rectangle. The proteins are superimposed based on the alignments between the rigid fragments. Protein nitrogen and oxygen atoms are shown as blue and red spheres, respectively, and water oxygens as red 3D “+” signs.

Several methods have been developed to compare binding sites and binding patterns (for example, refs. 19 and 65–67). As we are only interested in interactions with the adenine fragment, we developed ComBind (see Fig. 2) to detect polar (hydrogen-bonding) interactions of a given rigid ligand or a fragment of a ligand, both of which will be referred to as “fragments.” This software enables us to identify interaction patterns that are associated with the binding of specific fragments and that are shared or differ between protein families or folds that bind that same fragment.

### The Binding of the Adenine Fragment of Different Cofactors.

We examined the binding of proteins to the rigid adenine fragment within larger ligands. First, we used ComBind on the original dataset of Denessiouk and Johnson (9) and verified that our results were consistent with theirs (*SI Appendix*, Fig. S1). Their dataset included mainly proteins that bind adenosine triphosphate (ATP), with about 200 redundant proteins (from ∼500 complexes as some of the proteins have more than one structure in the PDB). Next, we compiled our own redundancy-reduced dataset of 985 PDB protein–cofactor complexes, in which any two proteins shared at most 30% sequence identity (68), and used ComBind to analyze the complexes. The cofactors in our dataset included the following cofactors and their analogs (*SI Appendix*, Fig. S2; the analogs are listed in *SI Appendix*, *Composing the datasets*): ATP (41% of the dataset), FAD (20%), SAM (17%), NAD (13%), and CoA (8%).

We detected the three motifs defined by Denessiouk and coworkers (9–11) in about half of the complexes in our dataset (Table 1), whereas the remaining complexes did not correspond to a specific predefined motif. The “reverse” and the “Asp” motifs were the two most common motifs in our dataset. In FAD-binding complexes, the “reverse” motif was the most prevalent (over 60%) whereas the “direct” motif was completely absent. In SAM-binding complexes, the “reverse” motif was observed in 17% of the complexes, and the “direct” motif was almost completely absent. In NAD- and CoA-binding complexes, both the “direct” and the “reverse” motifs were relatively rare. The “Asp” motif was more common than originally reported; it was identified in 36% of the NAD complexes and in 60% of the SAM complexes. In addition, we found this motif to be more variable than described; position II can be populated also by serine and cysteine residues, and not just by Asx/Glx as previously suggested. These variations were most common in SAM complexes. When considering the “Asp”’ motif in all its variations, we identified complexes containing this motif for each of the adenine-containing cofactors in our dataset, except CoA. The “direct” motif was the least common among the three motifs and was observed mostly in ATP-binding complexes. In these complexes, the “direct” and “reverse” motifs were observed in about 37% of the complexes, in almost equal numbers.

**Table 1.** Counts of the “direct,” “reverse,” and “Asp” variations of the adenine-binding motif, found in the dataset

Cofactor	The “direct” motif occurrence	The “reverse” motif occurrence	The “Asp” motif occurrence	Other	Total
ATP	77 (19%)	74 (18%)	10 (2.5%)	245 (60%)	406
FAD	0	128 (63%)	6 (3%)	69 (34%)	203
SAM	1 (0.5%)	29 (17%)	107 (61.5%)	37 (21%)	174
NAD	0	3 (2.5%)	42 (34%)	78 (63.5%)	123
CoA	2 (2.5%)	0	0	77 (97.5%)	79
					
Total	80	234	165	506	985

The total number (and percentage) of complexes of each type with the motifs are listed.

**Fig. 3.**
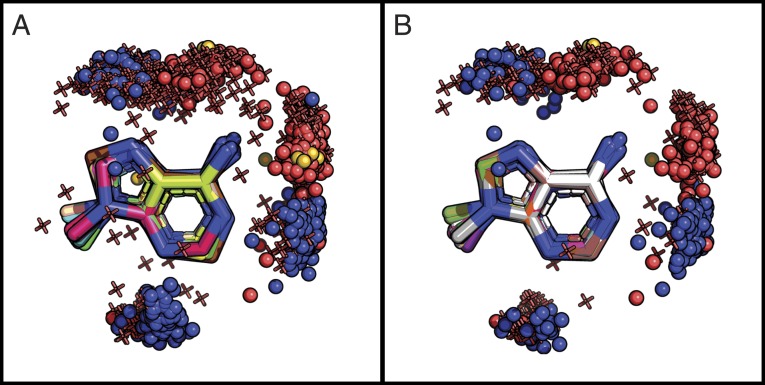
All of the hydrogen donors and acceptors of adenine are used for protein binding. (*A*) The hydrogen-bonding interactions with adenine for a dataset of 985 ATP-, NAD-, FAD-, SAM-, and CoA-binding proteins. Adenine carbon atoms are in various colors as each of them originates from a different crystal structure. Protein oxygen atoms are shown as red spheres, nitrogen atoms are shown as blue spheres, sulfur atoms are shown as yellow spheres, and water oxygen atoms are shown as red 3D “+” signs. (*B*) The same as *A*, focusing on the 406 ATP-binding proteins. The PyMOL sessions are given at http://trachel-srv.cs.haifa.ac.il/∼trachel/AdenineBinding/.

Importantly, for each of the three edges of adenine, we identified proteins that hydrogen-bonded to that edge (see Fig. 3*A*); this finding contradicts prior studies suggesting that proteins bind only to the Watson–Crick edge (refs. 9–11). For example, our analysis identified two conserved interaction clusters of the binding protein and adenine’s N6 group, one forming the known interaction with the Watson–Crick edge and the other forming another interaction with the Hoogsteen edge.

### Adenine Binding within the Context of ATP.

ATP-binding proteins were the most prevalent in our dataset (406 complexes) (Table 1) and spanned about a dozen different folds of the Evolutionary Classification of Protein Domains (ECOD) database (46). Because different protein folds might be characterized by different interactions with the adenine fragment of ATP, we used ComBind to characterize the geometry and interaction patterns of these proteins’ adenine interaction sites. As in our analysis of the full dataset of adenine-binding proteins, here, too, we observed a tendency of the proteins to exploit the full hydrogen-bonding potential of adenine (i.e., to bond on all three edges) (Fig. 3*B*).

To compare the binding sites of the different ATP–protein complexes, we carried out the following procedure. First, for each ATP-binding protein in the dataset, we identified the atoms in the protein that participate in equivalent hydrogen bonds with the adenine fragment. This set of atoms was referred to as the protein’s “interaction site.” We then constructed a network to visualize the relationships—i.e., the geometric similarities—among the interaction sites identified in the various ATP-binding complexes. In that network, each node represented an interaction site. We only included an interaction site in the network if it had at least three hydrogen bonds with adenine; 238 (59%) of the interaction sites satisfied this condition. We set this condition for inclusion in the network because our goal was to compare the geometry of the various interaction sites; at least three interactions are necessary to anchor the rigid adenine to the protein. Moreover, our ultimate aim was to identify evolutionary patterns so the capacity to take multiple hydrogen bonds into account when comparing two sites would enable us to identify more conserved patterns. Two nodes in the network were connected by an edge if they were “similar,” meaning that, when the adenine fragments of the complexes were superimposed, at least 60% of the atoms in the two interaction sites were the same (i.e., at least two out of three, three out of four, etc.), and the rmsd of their hydrogen-bonding partners was lower than 0.3 Å. Out of the total 238, 186 (78%) had another complex with a “similar” interaction site (i.e., were not singletons in the network).

**Fig. 4.**
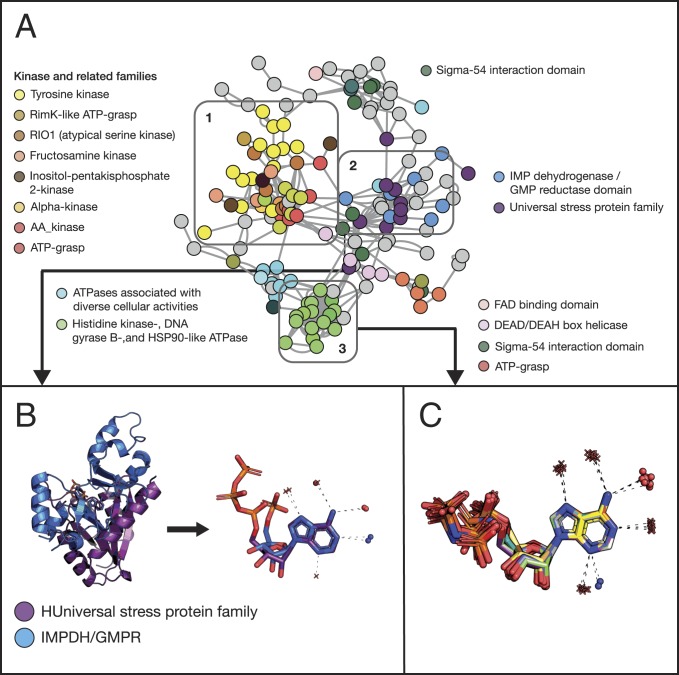
Adenine-binding patterns show complex relationships between protein sequence, structure, and function. (*A*) Proteins of similar families and function tend to form similar adenine-binding patterns. The nodes in the network represent protein–ATP complexes; edges connect two complexes with similar geometry in their interaction regions. Nodes are colored by the binding protein’s PFAM family; to reduce the color clutter, we assigned colors only to families with more than three complexes (the rest of the complexes are shown in gray), and we assign similar colors to related families (e.g., various kinases, as shown in rectangle 1). Proteins with very different sequences and structures can nevertheless have similar adenine-binding patterns. Rectangle 2 highlights such a cluster formed by the PFAM families: “Universal stress protein family” (in purple) and “IMPDH/GMPR” (in light blue, see *B* for details). (*B*) The *Left* of the panel shows two proteins, one from each of these families (PDB 3fdx in purple and PDB 3lfr in blue), with different global structures, aligned by their adenine fragment. The *Right* of the panel focuses on the adenine and the atoms that hydrogen-bond to it. Protein nitrogen and oxygen atoms are shown as blue and red spheres, respectively, and water oxygen atoms as red “+” signs. The dashed lines represent the hydrogen bonds. (*C*) Water molecules can be conserved in adenine binding: focusing on the complexes in rectangle 3 in *A*, we show a cluster of 16 complexes formed by PFAM family “Histidine kinase-, DNA gyrase B-, and HSP90-like ATPase”.

The largest connected component in this network had 151 interaction sites and is shown in Fig. 4*A*. The colors of the nodes encode the ECOD F-group classification (45), which is equivalent to PFAM’s protein family assignment (46). The figure further reveals that proteins from the same family (i.e., with similar sequences) tend to cluster together. This is expected as the sequence determines structure, including that of the binding site, and the binding site determines the interaction site. We also see that families of similar function cluster together (the same ECOD H-group—equivalent to SCOP’s superfamily)—meaning that they share similar adenine-binding modes. Protein kinases provide a good example of this phenomenon: We observe that the adenine-interaction-site clusters of several kinase groups are all connected (Fig. 4*A*, rectangle 1).

Interestingly, we also observed instances in which proteins with different sequences, folds, and even binding site topologies still share similar adenine interaction sites and even use the same binding motif. For example, the “universal stress protein” and “IMPDH/GMPR” PFAM families share only about 15% pairwise sequence identity and adopt different overall structures but nevertheless use the “reverse” motif and water molecules forming the same hydrogen bonds to bind adenine (Fig. 4*A*, rectangle 2, and Fig. 4*B*). These similarities suggest that the two protein families may have independently converged to similar forms of adenine binding or that the binding site “hopped” between these families.

We carried out several tests to evaluate the sensitivity of the results to our choices of tools and parameters. We observed that, while the details vary, the overall trends remain the same. In our main analysis, we relied on the hydrogen-bond definitions used by Arpeggio (69); *SI Appendix*, Table S1 shows that these agree with the bonds identified through other commonly used tools. *SI Appendix*, Fig. S4 shows ComBind’s results for the dataset of ATP-binding sites with different distance thresholds for hydrogen bonds (3.9 Å and 3.2 Å); with these thresholds, too, proteins used all three edges of adenine for binding (*SI Appendix*, Fig. S4 *A* and *B*), and adenine-binding patterns tended to be similar among proteins of the same family (*SI Appendix*, Fig. S4 *C* and *D*). The two parameters used when comparing two sites—the rmsd and the percent of interacting atoms in close proximity—also influenced our results: Using laxer thresholds resulted in a larger number of binding sites being identified as “similar” to each other, making the network more connected and rendering it harder to trace the clusters (*SI Appendix*, Fig. S4*E*). Using stricter thresholds disintegrated the network into numerous connected components (*SI Appendix*, Fig. S4*F*). Nonetheless, the formation of clusters of protein families sharing the same adenine-binding patterns remained. Thus, we carried out our main analysis using thresholds that were relatively strict (low rmsd) to ensure that equivalent hydrogen bonds would be detected, but not so strict that we could not see any relationships among more remote binding sites.

### Adenine Binding and Themes.

The observations outlined above suggest complex relationships between sequence, structure, and function within adenine-binding sites. To further explore these relationships, we studied the themes (evolutionary building blocks) that construct the binding sites. Specifically, we looked for themes that participate in the binding; a theme participates if at least one of its residues hydrogen-bonds to the adenine fragment of the ligand [see discussion above and the work of Nepomnyachiy et al. (13), which introduced this concept]. *SI Appendix*, Fig. S5 shows the multiple sequence alignment (MSA) of the nine segments from seven proteins that form one theme (number 1403), alongside the segments shown in color within the context of their chains’ sequences and structures.

**Fig. 5.**
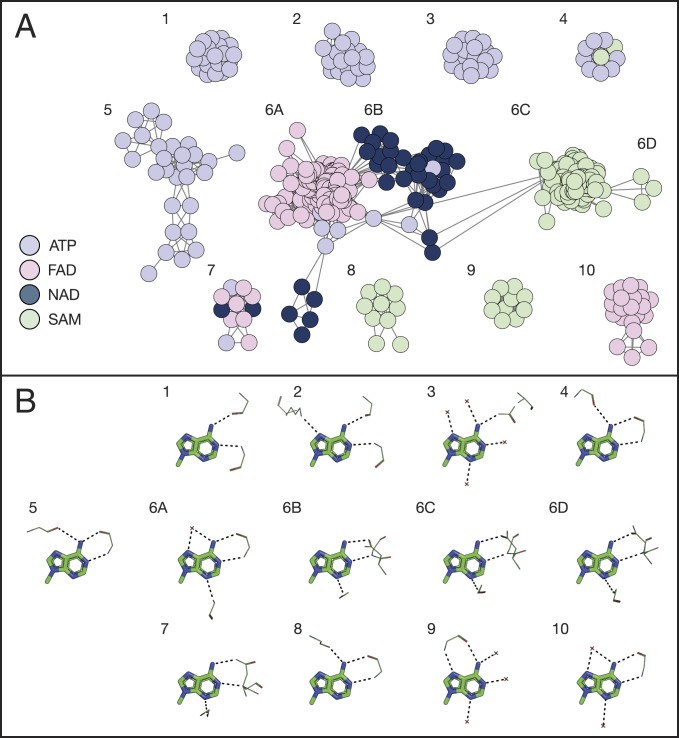
A network representation of themes in nucleotide cofactor-binding proteins shows that themes represent distinctive binding modes. (*A*) Proteins use different themes to bind different ligands. Each node in the network represents a protein–ligand binding site. The nodes are colored according to the bound ligand, following the color legend at the bottom left of the panel. Connected nodes have at least one theme in common in the binding site. (*B*) A representation of the binding modes related to the clusters. The numbering of the binding modes corresponds to the cluster number in *A*. Only the adenine fragment is shown; protein atoms are shown in lines representation, and side chains are shown only when mediating binding. The hydrogen bonds with adenine are shown as black dashed lines. The interacting atoms around adenine show the consensus binding mode of the cluster.

**Fig. 6.**
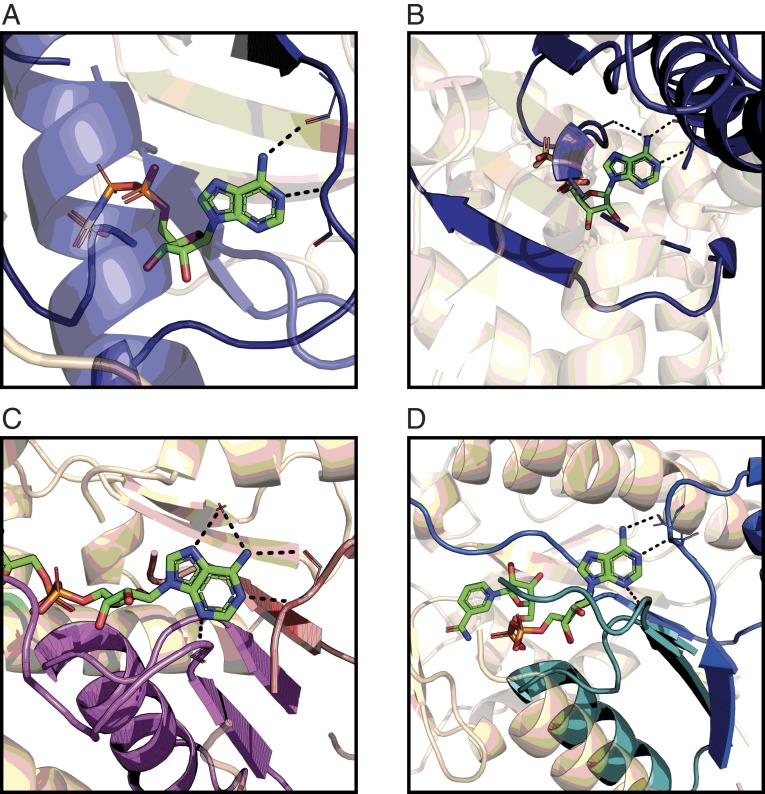
Themes can form the scaffolds for adenine binding in proteins. The protein is shown in wheat with the themes highlighted in colors. The adenine-containing ligand is shown using a bond-stick model, and the hydrogen bonds to specific amino acids of the themes and water molecules are in black dashed lines. (*A*) A theme representing the “direct” motif in ATP binding, as found in Fig. 5*A*, cluster 1 (demonstrated using PDB 5ckw). (*B*) A theme representing the “reverse” motif in ATP binding, with an additional interaction between the protein and adenine’s N6 in the Hoogsteen edge, as found in Fig. 5*A*, cluster 4 (demonstrated using PDB 1g41). (*C*) A combination of two themes (purple and pink) composing adenine’s binding site in FAD, as found in Fig. 5*A*, cluster 6A (demonstrated using PDB 2gag). (*D*) A combination of two themes (blue and cyan) composing adenine’s binding site in NAD, as found in Fig. 5*A*, cluster 6C (demonstrated using PDB 5u4q). The PyMOL sessions are given at http://trachel-srv.cs.haifa.ac.il/∼trachel/AdenineBinding/.

We looked for a recurrence of themes in our dataset of protein–adenine complexes (for more details, see *SI Appendix*, *Composing the theme dataset for adenine binding proteins*). In Fig. 5, the similarities among the identified themes are visualized through a network. Each node represents a protein–adenine complex, and edges connect nodes whose protein part share a theme (Fig. 5*A*). Specifically, we added an edge when the same theme appeared in both complexes, and, in each theme, at least one amino acid hydrogen-bonds the adenine. The nodes are colored according to the bound ligand. The figure shows that proteins that share the same theme tend to bind the same cofactor. Moreover, when we examine all of the large network clusters that comprise 10 or more nodes (the full network includes 37 clusters, 10 of which are “large”), we see that each corresponds to a distinctive binding pattern of adenine (Fig. 5). The participation of themes in adenine’s binding mode is briefly summarized below and demonstrated in Fig. 6 (a detailed description of binding patterns and themes associated with each cluster is given in *SI Appendix*).

#### Clusters of ATP-binding proteins.

Our analysis of common themes revealed five large clusters of ATP-binding complexes (Fig. 5*A*, clusters 1 to 5), primarily differing in the themes they use for adenine binding. This observation translates into binding patterns that are cluster-specific (Fig. 5*B*). For example, the binding pattern of cluster 1 is the “direct” motif (Fig. 6*A*) whereas all of the complexes of cluster 4 use the “reverse” motif, with an additional interaction between the protein and adenine’s N6 in the Hoogsteen edge (Fig. 6*B*). We find another cluster (Fig. 5*A*, cluster 4), formed entirely by proteins from PFAM’s IMPDH/GMPR family, which shows a variation on previously known motifs. All of the proteins in the cluster bind adenine in the Watson–Crick edge using the “reverse” motif, but they also have an additional interaction between a backbone carboxyl group at “position XV/XVI” (22 of 23 residues downstream to the residues forming the reverse motif) and adenine’s N6 in the Hoogsteen edge (*SI Appendix*, Fig. S3*A*).

#### Clusters of other nucleotide-binding proteins.

Clusters 6 to 10 in Fig. 5*A* show additional interesting patterns. First, we see how a combination of themes can be used to form a complete interaction site. Cluster 6, with 242 complexes, can be divided into several connected subclusters and is formed mostly by proteins that ECOD classifies within their Rossmann-like X-group. The binding sites in the FAD-binding subcluster (Fig. 5*A*, cluster 6A) use a combination of two cooccurring themes to bind adenine (Fig. 6*C*). Proteins in the NAD-binding subcluster (Fig. 5*A*, cluster 6B) use a combination of three themes to bind adenine (Fig. 6*D*), but not all three are required to form the binding site (meaning, we see a “mix and match” of these three themes). Some of the clusters exhibit new adenine-binding motifs. For example, the proteins in a cluster formed completely by SAM-binding proteins in PFAM’s SET domain family bind adenine in a pattern similar to the “reverse” motif except that the interactions are between the backbone amide and carboxyl group of the amino acid in “position III” and adenine’s N6 in the Hoogsteen edge and N7. There are additional hydrogen bonds between the protein and other adenine atoms, but this new “motif” is the only one shared by all of the proteins in the cluster (Fig. 5 *A* and *B*, cluster 9, and *SI Appendix*, Fig. S3*B*).

### Adenine-Binding Themes in the PDB.

Our analysis detected themes that tend to be a part of adenine-binding sites. However, the participation of these themes in adenine binding does not necessarily mean that they were selected during evolution for this task. To estimate the degree of “dedication” of the themes in Fig. 5*A* to adenine binding, we searched for them in the entire PDB. We found that 47% of the PDB entries that contain these themes also include adenine-containing ligands. Since these ligands appear in only 9% of the entire PDB, our results indicate that adenine-binding proteins are enriched with the themes in Fig. 5*A*. Furthermore, about half of the PDB entries, which contain an adenine-binding theme but no ligand, share at least 80% of their sequence with those that do contain adenine in their structure. The latter observation could indicate that, although the structures of these proteins were determined without a bound ligand, their biological function requires interaction with an adenine-containing ligand. This analysis further supports the evolutionary link between the themes and adenine binding. Encouraged by these results, we proceeded to analyze all entries in UniProt for adenine-binding themes and detected them in about 4% (1,144,830) of the sequences (70) (Dataset S2).

## Discussion

Here, we studied protein–adenine interactions and investigated how specific recurring sequence segments in adenine-binding proteins—namely, “themes,” which are proposed to serve as evolutionary building blocks—relate to the patterns that proteins use to bind the adenine fragment of different nucleotide cofactors, as well as to biological function.

We relied on a large, comprehensive, and nonredundant dataset of protein–nucleotide complexes from the PDB. The quality of the structures in the dataset varied, with resolutions ranging from 0.74 to 6.93 Å (but only 31 structures had resolutions worse than 3 Å), and R-scores ranging from 0.117 to 0.341. More than 75% of structures had resolutions of 2.5 Å and R-scores of 0.25 or better. Our analysis was constrained by the information available in the PDB, which is biased due to the methodological constraints and research interests of the contributing experimentalists (71). However, we trust our observations for two reasons: 1) The same main results were observed in preliminary analyses of other datasets; and 2) the interactions that we report are present in multiple structures, making an error less likely. It is possible, however, that our analysis failed to detect some interactions that were present, owing to insufficiently high resolution of the complexes in the PDB. In addition, the results are sensitive to the geometric definitions of hydrogen bonds, a known problem that has been discussed extensively elsewhere (24–26). The results reported herein were obtained using the default parameters suggested by Arpeggio (69); we reran our analysis using alternative definitions of hydrogen bonds and obtained the same main results (*SI Appendix*, Fig. S4 *A*–*D*).

Our analysis did not account for additional types of possible noncovalent interactions between the protein and the adenine moiety. Of these, aromatic interactions are expected to be important and common—both because adenine is made of aromatic rings and because aromatic interactions include both electrostatic and nonpolar components, which, along with the planar nature of the rings, contribute to the specificity of the binding. We found that such interactions appeared in one-third of the complexes. However, they were evenly distributed between the different cofactors and did not form any specific patterns.* This suggests that the main role of these interactions was to provide geometric complementarity for the binding, rather than specificity, and, as such, they are less likely to offer insight regarding the evolution of molecular recognition.

Previous studies revealed interactions of proteins with the Watson–Crick edge of the adenine fragment and did not identify interactions with adenine’s other edges, despite the availability of hydrogen-bonding opportunities on all edges. These findings effectively implied that all cases of adenine binding diverged from a single evolutionary event. Our results, based on a fivefold larger (albeit redundancy-reduced) dataset, confirm the interaction patterns proposed by previous studies and reveal additional patterns. We find that proteins interact with all three edges of the adenine fragment, suggesting that adenine binding emerged several times in evolution—a phenomenon that may be indicative of convergent evolution.

On the protein side of the interactions, we identified the interaction motif suggested by Denessiouk and coworkers, which they subdivided into “direct,” “reverse,” and “Asp” variants, based on the relative positions of the interacting amino acids (10, 11). We identified these motif variants in the binding sites of all adenine-containing cofactors that were considered by Denessiouk and coworkers (9–11). However, we observed that, contrary to their findings, the most common variation is the “Asp” motif, which was found in the binding sites of almost all of the cofactors in our dataset. We also found that, in many cases, water molecules mediate binding interactions between groups that are too far from one another to interact directly, and, in some cases, replace amino acids as hydrogen bond donors and acceptors. Since we observed protein groups or water molecules in equivalent positions that interact with the ligand (e.g., Fig. 3), it could be that these water molecules are conserved in certain types of complexes. Indeed, we identified conserved water molecules in multiple different adenine-binding families (e.g., Fig. 4*C*). The conservation of water molecules within protein-binding and catalytic sites has been documented in many cases and has even been linked to the protein’s function (72, 73).

Our analysis of ATP-binding proteins shows that proteins with similar sequences often share similar binding geometry, but also that similar binding geometries may appear in proteins with very low sequence and structure similarity. This finding might suggest that ligand-binding patterns in proteins, as reflected by the interaction site and binding mode, are more conserved than the overall sequence and structure; alternatively, it might suggest that a given ligand-binding pattern can arise independently on multiple occasions. In support of the latter, the protein of known structure (55) of the Keefe and Szostak designs (54) does not contain any of our themes—which is not surprising as it does not share an evolutionary origin with the adenine-binding proteins in our dataset. Nonetheless, its adenine-binding mode closely resembles the one shared by proteins in cluster 6A in Fig. 5 (*SI Appendix*, Fig. S6), suggesting that nature has exhausted all of adenine’s binding modes.

Ligand-binding patterns in proteins are determined by the structures and amino acid compositions of the proteins’ binding and interaction sites. To better characterize the determinants of the adenine-binding interactions and potentially to gain broader insights into protein evolution, we focused on the binding sites’ evolutionary building blocks. The concept of themes, as proposed by Nepomnyachiy et al. (13), challenges the widespread notion that only domain-level units constitute the evolutionary and functional building blocks of proteins; it suggests that evolutionary units may be much smaller than domains. The current study provides support to this suggestion by demonstrating that certain themes tend to appear in nucleotide-binding sites (and interaction sites) where they participate directly in the binding. Specifically, we obtained the following findings: First, certain themes are associated with the binding of specific nucleotide ligands (Fig. 5), and their presence in a given protein predicts with high certainty that this protein binds adenine. Second, most of the themes interact with nucleotide ligands in a specific way, and many of the themes constitute parts of known nucleotide-binding motifs (Fig. 6 *A* and *B*) or even extend them (e.g., clusters 5 and 9 in Fig. 6 and *SI Appendix*, Fig. S3 *A* and *B*). Third, certain theme combinations tend to appear together in interaction sites (Fig. 6 *C* and *D*). Again, this further supports the notion of themes acting as evolutionary building blocks of proteins. The current study connects specific themes, identified on the basis of sequence recurrence across proteins, to a molecular function. Our results show that proteins containing certain themes are about five times more likely to bind adenine than a random protein in the PDB and predict that about 4% of UniProt’s proteins may bind adenine. This finding demonstrates how themes can be used in function prediction. It is also noteworthy that combinations of themes that often recur in the binding sites of different proteins form the same three-dimensional (3D) geometry and the physicochemical environment required for the binding.

## Conclusions

This study focused on two key aspects of protein–adenine interaction: 1) the physicochemical nature of the binding, as reflected by the hydrogen-bonding interactions between the protein and ligand and by the structural motifs that support this binding; and 2) the evolutionary traces related to binding, as reflected by the role of highly reused sequence “themes.”

Adenine offers many hydrogen donors and acceptors on the Watson–Crick, Hoogsteen, and sugar edges; our analysis shows that proteins may take advantage of various combinations of these, demonstrating the opportunistic nature of evolution. In addition, we correlated this knowledge with evolutionary trends in adenine-binding proteins, as reflected by their theme composition. The different geometries and binding patterns found in various proteins suggest that adenine binding emerged more than once in evolution, pointing to convergent evolution. Future efforts toward deciphering the emergence of molecular recognition can be directed toward creating an evolutionary path that underlies the gradual construction of larger themes, and of related binding sites (and hence, interaction sites), from an initial finite set of short themes.

On a broader level, our results support the idea that themes are not merely recurrent protein sequences but (at least in some cases) are conserved functional units. It is tempting to suggest that ligand binding has emerged from combinations of such functional building blocks: segments of varied sizes, which, due to their ability to form interactions with the ligand, were conserved and coupled together to form larger interaction sites. Although the proteins in our dataset share at most 30% sequence identity and adopt different folds, many of them use the same themes to bind adenine. This would be consistent with two hypothetical (and complementary) evolutionary scenarios. According to the first, various proteins have evolved around a primordial adenine-binding theme, and, according to the second, adenine-binding themes have migrated among proteins of various folds.

The procedure that we introduced here can be used to study the binding of rigid fragments of any ligand. In addition, it can be used to explore themes that are involved in other molecular functions (e.g., catalysis) and thus to contribute to the construction of a functional “theme vocabulary.” Such a vocabulary can assist researchers in assigning functions to newly discovered proteins, as shown in our UniProt predictions (Dataset S2), or in identifying binding sites, as we find potential adenine-binding sites in the PDB. In addition, a functional theme vocabulary can improve methods of molecular docking and add functionalities to designed proteins. For example, the “direct” and “reverse” variants of the adenine-binding motif use backbone interactions to bind the adenine fragment, making it difficult to use these variants for protein design purposes. However, the observation that specific adenine-binding themes might produce these variants suggests that it might be possible to incorporate these themes into designed proteins and thus to obtain a desired motif more easily. In this way, a theme vocabulary may be used to approach difficult challenges in protein design.

This work links themes to a well-defined biological function, thereby shedding light on the relationships between the sequence, structure, and function of the evolutionary building blocks forming proteins. We hope it paves the way to future works in this field, which will ultimately help scientists reveal how the complex protein universe has emerged.

## Materials and Methods

The ComBind pipeline is implemented in Matlab and includes a few steps. Briefly, for a given query fragment, it searches the PDB for all instances of ligands that contain that fragment; to this end, it uses planar representations of the ligands. Then, ComBind downloads all of the PDB entries containing these ligands and superimposes their 3D structures on the query fragment (Fig. 2). Next, ComBind uses Arpeggio (69) to identify polar interactions between the fragment and its interaction site in each of the PDB entries. Finally, ComBind creates a PyMOL (74) session containing the fragment and the atoms of the interaction sites (Fig. 2). For more details, see *SI Appendix*.

### Data Availability.

ComBind code can be found on https://bitbucket.org/ayanarun/combind/src/master/.

## Supplementary Material

Supplementary File

Supplementary File

Supplementary File

## Footnotes

*The PyMOL session displaying the interactions is at http://trachel-srv.cs.haifa.ac.il/∼trachel/AdenineBinding/.
