# Supplementary material for: On the evolution of protein–adenine binding
Source: Proc Natl Acad Sci U S A. 2020 Feb 20;117(9):4701–9. doi: 10.1073/pnas.1911349117 (PMC7060716; doi:10.1073/pnas.1911349117)
Supplement: Supplementary File [file pnas.1911349117.sapp.pdf]

PNAS
www.pnas.org

Supplementary Information for

On the evolution of protein-adenine binding

Aya Narunsky[1], Amit Kessel[1], Ron Solan[1], Vikram Alva[2], Rachel Kolodny[3*], Nir Ben-Tal[1*]
[1]Department of Biochemistry and Molecular Biology, George S. Wise Faculty of Life Sciences, Tel Aviv University, 69978 Ramat Aviv, Israel
[2] Department of Protein Evolution, Max Planck Institute for Developmental Biology, 72076 Tübingen, Germany
[3] Department of Computer Science, University of Haifa, Mount Carmel, 3498838 Haifa, Israel
Nir Ben-Tal
Email: bental@tauex.tau.ac.il
Rachel Kolodny
Email: trachel@cs.haifa.ac.il

This PDF file includes:

Supplementary text
Figures S1 to S6
Legends for Datasets S1 to S2
SI References

Other supplementary materials for this manuscript include the following:

Datasets S1 to S2

Supplementary Information Text

Clusters in the network of adenine-binding regions. There are 37 clusters in the adenine-binding network, containing overall 528 nodes. Our analysis only focuses on clusters with 10 or more nodes, resulting in 10 clusters with 388 nodes. In four of these clusters water molecules are used for coordination with the adenine fragment.

Themes found in adenine-binding regions in proteins. There are 756 themes found in adenine-binding regions. Of these, 537 are found in more than one binding region, and so connect two protein-adenine complexes in the network. Figure 5A only shows the clusters with 10 or more adenine-protein complexes; 151 themes are shared by the complexes forming the network in the figure.

Clusters of ATP-binding proteins. There are five large ATP-binding protein clusters (Figure 5A, clusters 1-5). Each of the clusters represents a different adenine-binding pattern, as shown in Figure 5B. Two clusters bind adenine by the direct motif (as demonstrated in Figure 6A), with one of the clusters including an additional interaction between the protein and N7 of adenine. The themes shared by proteins in this cluster are involved in either of these interactions, or in both. The third cluster, formed entirely by P-loop proteins, represents the reverse motif, usually with an additional interaction involving N6 of adenine, in the Hoogsteen edge (Cluster 5 in Figure 5 A and B, also demonstrated in Figure 6B). The fourth cluster, formed entirely by proteins from PFAM's IMPDH/GMPR family, shows an interesting variation on previously identified motifs. All the proteins in the cluster bind adenine in the Watson-Crick edge using the reverse motif, but they also have an additional interaction between a backbone carboxyl group at 'position XV/XVI' (22/23 residues downstream to the residues forming the reverse motif) and adenine's N6 in the Hoogsteen edge (Figure S3A). The complexes in the fifth cluster lack the direct or reverse motifs. Instead, a single backbone carbonyl group of the protein interacts with N6 of adenine, and in most of the complexes there are also interactions between water molecules in the binding site and adenine.

Clusters of other nucleotide-binding proteins. We examined five clusters: one included FAD-binding proteins, all of which bound adenine via the reverse motif (cluster 10 in Figure 5, A and B), and in many of them binding was mediated by water molecules. The second cluster mostly contains proteins that bind FAD, and most of the proteins in this cluster bind adenine via the 'Asp' motif, combined with another interaction between adenine's N3 and the backbone's amide group (Figure 5, A and B, cluster 7). The third and fourth clusters contain proteins that bind SAM: the proteins in the third cluster bind adenine via the reverse motif, often with an additional interaction between adenine's N6 in the Hoogsteen edge and a backbone carboxyl group (Figure 5, A and B, cluster 8). The proteins in the fourth cluster, all belonging to PFAM's SET domain family, bind adenine in a pattern that seems similar to the reverse motif, except that the interactions are between the backbone amide and carboxyl group of the amino acid in 'position III' and adenine's N6 in the Hoogsteen edge and N7. There are often additional interactions between the protein and other adenine atoms, but this 'motif' is the only one shared by all the proteins in the cluster (Figure 5, A and B, cluster 9, and Figure S3B). The fifth cluster can be divided into four interconnected sub-clusters (Figure 5, A and B, cluster 6), with one sub-cluster binding FAD, one sub-cluster binding SAM, and two interconnected sub-clusters binding NAD. The complexes in each sub-cluster bind adenine differently, and use different themes for this purpose, as follows:
The proteins in the FAD-binding sub-cluster use a combination of two themes to bind adenine (Figure 6C): one forms an interaction via the reverse motif, and the other adds an interaction between the protein's backbone and adenine's N3. In addition, we see a conserved interaction with water molecules and N7 of adenine.

The proteins in the NAD-binding sub-cluster use a combination of three themes: one forms an interaction via the ”Asp” motif, the second adds an interaction with N3 of adenine, and in some cases a third theme adds an interaction with N6 of adenine in the Hoogsteen edge (Figure 6D).
The proteins in the SAM-binding sub-cluster use a relatively long theme to bind adenine: This theme forms an interaction via the ”Asp” motif and adds an interaction with N3 of adenine. In addition, we see a conserved water molecule interacting with N6 of adenine in the Hoogsteen edge and with N7 of adenine (Figure S3C).

Themes used in adenine binding. In the following section we list all the themes shared by the clusters in our dataset. The numbering of the clusters follows the scheme in Figure 5.

<u>Cluster #1:</u>
The cluster is composed of 20 binding sites; the vast majority of them (18) bind adenine via the direct motif, as described by Denessiouk and co-workers (1-3). The themes shared by proteins in the cluster are numbered 867, 873, 896, 1354, 1355, 1356, 1357, 1648, 1924, 2421, 2422, 2423, 2527.

<u>Cluster #2:</u>
The cluster is composed of 20 binding sites; almost all of them (18) bind adenine via the direct motif, and in addition, most of the binding sites (16) have an additional interaction between adenine and the amino group of a lysine residue with adenine’s N7. The themes shared by proteins in this cluster can be divided into three groups: themes that cover the entire binding pocket (includes themes 226, 227, 229, 230, 393, 395, 858, 859, 861, 126, 1327, 1328, 1384, 1389, 2451, 2457), themes that represent the direct motif (includes themes 232, 1325, 1384, 1385, 1388, 1392, 2332, 2416, 2417, 2419, 2452, 2453), and a single theme shared by almost all of the binding sites that include the additional N7/lysine interaction (228).

<u>Cluster #3:</u>
The cluster is composed of 18 binding sites; in all of them adenine’s N6 in the Watson-Crick edge binds to the carboxylate group of an aspartate residue, and the rest of the interactions of adenine and its environment are with water molecules. The themes shared by proteins in this cluster donate this aspartate (themes 21, 23, 35, 289, 290, 292, 659, 1007).

<u>Cluster #4:</u>
The cluster is composed of 11 binding sites, binding either ATP (or its analogs) or SAM using the reverse motif. In addition, all of them have an additional interaction between adenine’s N6 in the Hoogsteen edge and a backbone carboxyl group at ‘position XV/XVI’ (22/23 residues downstream to the residues forming the reverse motif, Figure S3A). The themes shared by proteins in this cluster usually form the full binding region (themes 1403, 1405, 1406, 1455, 1458, 1460, 1753, 1754, 1755, 2061, 2095), or only position XV/XVI (themes 1404, 1456, 1457, 1459, 2060).

<u>Cluster #5:</u>
The cluster is composed of 28 binding sites and can be roughly divided into three connected components connected in a linear order. Generally speaking, the proteins in the cluster bind adenine mostly via the reverse motif, with an additional interaction of adenine’s N6 in the Hoogsteen edge. This binding mode is especially common amongst the proteins in the center of the cluster.

<u>Cluster #6</u>
This interesting cluster contains 241 binding sites and can be divided into several sub-clusters, each containing a different ligand’s binding sites: SAM, NAD and FAD. For the SAM and NAD, the dominate binding mode is usually the ”Asp” motif, where adenine’s N6 in the Watson-Crick edge hydrogen bonds to the carboxylate group of an aspartate residue, with an additional interaction between the protein and adenine’s N3 (for SAM) or N6 in the Hoogsteen edge (for NAD). For FAD, the most common binding mode involves the reverse motif, with an additional interaction between the protein and adenine’s N3.

<u>Cluster #6A</u>
A large cluster, composed of 113 nodes; most of them represent FAD-binding proteins. The vast majority of the proteins in the cluster (104) bind adenine using the reverse motif. In addition, we

noticed an additional interaction with adenine's N3 in 103 of the proteins in the cluster, and interactions with the rest of adenine's binding atoms with water molecules in its environment. The themes shared by proteins in this cluster are divided into two groups, according to the binding mode they represent: the first group contains themes representing the reverse motif (themes 39, 40, 46, 320, 332, 333, 1055, 1200, 1202, 1219, 1220, 1221, 1454, 1663, 1683, 1734, 1921, 1968, 2086, 2329), while the second group donates backbone interaction with adenine's N3 (themes 331, 335, 365, 61, 981, 1919). In addition, we noticed less common themes, representing interaction with adenine's N7 (themes 461 and 561).

Cluster #6B

The cluster is characterized by the ”Asp” motif (found in 9 of the 10 proteins in the cluster), but with an additional interaction of adenine's N3 with the protein (or with a water molecule, in three proteins), and in some cases, interactions of adenine's N6 in the Hoogsteen edge either with the residues of amino acids or with water molecules. The themes in the cluster can be divided into three groups: a theme involved in the ”Asp” motif (theme 623), a theme involved in binding of adenine's N3 (theme 279), and themes involved in the binding of adenine's N6 in the Hoogsteen edge (themes 101, 498, 648, 831, 832, 962).

Cluster #6C

This cluster is mainly characterized by the variation of the direct motif, as described in cluster #5A, which is found in 15 of the 17 binding sites composing the cluster. Themes shared by proteins in the cluster indeed represent this binding mode (themes 100, 281, 977, 1363).

'Bridging nodes' between cluster #6C and cluster #6D

There are three nodes connecting the cluster of SAM-binding proteins with NAD-binding proteins. Two of the nodes represent NAD binding sites; the third node represents an ATP binding site. For the two NAD-binding proteins, the theme connecting the proteins to the SAM-binding proteins creates a large scaffold for the entire binding of adenine (theme 944). It has some overlap with the themes connecting them to the cluster of NAD-binding proteins (theme 949). For the ATP-binding protein, the same theme (theme 944) has smaller coverage; hence it is shorter. However, it includes an interaction between a backbone amide group and adenine's N3, and the variation of the direct form of the adenine-binding motif as described in cluster #6D.

Cluster #6D

The cluster is composed of 81 nodes; the vast majority of them (66) bind adenine in the ”Asp” motif, where N6 in the Watson-Crick edge hydrogen-bonds either to the carboxylate group of aspartate, to the amide group of asparagine, or to the hydroxyl group of serine. In addition, most of the binding sites in this cluster (59) have an additional interaction, where adenine's N3 hydrogen-bonds to the amide group of another residue, which is found 25-30 amino acids upstream to the amino acid binding N1. The themes shared by proteins in this cluster can be divided into three groups: themes that create the 'scaffold' of the binding (themes 186, 793, 1756), a theme that donate the interactions between the protein and the Watson-Crick edge of adenine (318), and themes that donate the interaction with adenine's N3 (31, 317, 631).

Cluster #7:

This cluster, with 11 binding sites in total, is composed mainly of FAD binding sites, but also includes two ATP binding sites and two NAD binding sites. Most of the binding sites (8) bind adenine via the ”Asp” motif, except for two binding sites using the reverse motif, and another binding site where the interaction with adenine's N6 in the Watson-Crick edge is mediated by a water molecule. Seven of the binding sites have an additional interaction between adenine's N7 and a backbone amide group, and six of the binding sites have an additional interaction between adenine's N6 in the Hoogsteen edge and a water molecule. The themes shared by the binding sites in this cluster donate the ”Asp” motif together with the interaction with adenine's N3 (themes 89, 92, 485, 488, 491, 492, 1020, 1023, 1025).

Cluster #8:

The cluster is composed of 10 SAM binding sites; 9 of them bind adenine via the reverse motif, and in 7 of them there is an additional interaction between adenine's N6 in the Hoogsteen edge and a

backbone carbonyl group (in another binding site this interaction is mediated by a water molecule). Themes shared by the binding sites in the cluster donate both interactions, in a large binding-site scaffold (themes 516), or only form the additional interaction with adenine's N6 (themes 2300, 2301, 2344).

Cluster #9:

This cluster is composed of 10 SAM-binding proteins, all of which belong to PFAM's 'SET' family. Their adenine-binding pattern is unique: it is very similar to the reverse motif, except here the interactions are between the backbone amide and carboxyl group of the amino acid in "position III" and adenine's N6 in the Hoogsteen edge and N7. The themes shared by proteins in this cluster form this motif (themes 1125, 1127, 1128, 1843, 1845, 2314, 2325). Other interactions that are found in this cluster are not represented by themes shared by proteins in the cluster.

Cluster #10:

The cluster is composed of 21 FAD-binding proteins, all of which bind adenine by the reverse motif. In all of them there is an additional interaction between adenine's N6 in the Hoogsteen edge, mostly with water molecules. In addition, in 17 of the binding sites there is an additional interaction between adenine's N7 and, in most cases, a water molecule. The themes shared by proteins in the cluster form the reverse motif (themes 254, 255, 256, 257, 776, 777, 778, 889, 895, 987, 1173 2166, 2169, 2170, 2179, 2397, 2398). It is noteworthy that many of these themes are quite long; some cover more than 100 amino acids.

Supplementary Methods

The ComBind methodology

2D representations of the ligands. 3D coordinates of the ligands are downloaded from the PDB, and their 2D representations are produced using the OpenBabel chemistry toolbox (4).

Identify the adenine fragment in a ligand. To identify the adenine fragment of a ligand, the distances (in Angstroms) between all the nitrogen atoms in a 2D representation of the ligand are calculated, and compared to the distances between adenine nitrogen atom pairs. When the algorithm identifies a pair of identical distances, it shifts the ligand to the adenine to match the two nitrogen atoms. Next, it uses the Hungarian algorithm (5) to find the ligand atoms that have the closest proximity to the adenine atoms, and calculates the RMSD between these atoms and adenine. The ligand is considered as containing adenine if this RMSD is small enough (less than 0.1Å). ComBind then uses the Kabsch (6) algorithm to calculate a rotation and translation that optimally superimposes the adenines on one another, and uses it to transform the bound proteins. ComBind can be used with any rigid fragment of ligands. The code can be found in http://bitbucket.org/ayanarun/combind/src/master/.

Identify hydrogen bonds between the adenine and its environment. The polar interactions between the adenine and its surroundings (e.g., water molecules and amino acids) are identified using Arpeggio (7). The ligand and the atoms that hydrogen-bond with the adenine are extracted to a PyMOL session, and this collection of atoms is referred to as the 'interaction site' of adenine.

Our focus on hydrogen bonds for characterizing binding patterns is motivated by the fact that such bonds, which are very common in proteins (8-11), are prevalent in ligand-binding sites and are important for the specificity of protein-ligand interactions (12-14). The role of such bonds in specificity is due in part to the dependence of their free energy on their geometry (i.e., on bond length and angles) (10, 15, 16). Moreover, hydrogen bonds are easier to identify compared with other protein-adenine interactions, such as those involving π electrons (π–π and cation-π interactions). The latter interactions, although specific in nature (17), are weaker than canonical hydrogen bonds, less common, and their energy dependence on chemical and geometric characteristics is much more complicated (18). Generally, these contribute to the affinity of the binding, while hydrogen bonding adds to its specificity (19-22). Aromatic residues are often found in ligand binding sites (23-26).

However, the interactions of these residues with the ligand include also non-specific hydrophobic and van der Waals components.

Composing the datasets

We collected all the proteins in the PDB that bind adenosine triphosphate (ATP) and its analogs (PDB ligands: ADP, AMP, ANP, ACP, DTP, AGS, DAT, APC, A12, AN2, ADX, M33), nicotinamide adenine dinucleotide (NAD) and its analogs (PDB ligands: 8NA, A3D, CNA, DND, NXX, NAP, NA0, NJP), flavin adenine dinucleotide (FAD) and its analogs (PDB ligands: 6FA, FAS, 5X8), S-adenosyl methionine (SAM) and its analogs (PDB ligands: SAH, SMM), and coenzyme A (CoA) and its analogs (PDB ligands: CAO, COS, COZ, 1VU, ACO, BCO, IVC, ACO). We selected analogs that did not change the functional part of the ligands, and where the adenine fragment remained unchanged. We removed redundancy from the dataset, selecting only the proteins sharing at most 30% sequence identity. Clustering was performed using the sensitive cluster mode of MMseq2 (27, 28) and at a length coverage of 70%. From the resulting clusters, one representative per cluster was chosen based on resolution, R-free factor, and completeness; when possible, crystal structures were preferred over NMR structures. The resulting dataset included 985 entries; 751 of them (76%) were structures of very good quality (resolution under 2.5Å, free-R value under 0.25), in 113 (11%) entries the resolution was between 2.5-6.93Å, and in the rest (121, 12%) of the structures the resolution was good (2.5Å or better) but the free-R value was between 0.25 and 0.3.

Network of binding patterns

To compare the adenine interaction sites in two different complexes, the closest binding site atoms were detected using the Hungarian algorithm. After the matching atoms were detected, the RMSD between them was calculated using the Kabsch (6) algorithm. We considered two interaction sites as 'similar' if this RMSD was under 0.3Å, and the corresponding atoms included at least 60% of the atoms in each of the interaction sites. We performed this calculation for all vs. all adenine interaction sites in our ATP datasets, and used Cytoscape (29) to visualize the network (Figure 4). Each node in the network represents an ATP (or analog)-interaction site, where the adenine fragment has at least 3 hydrogen bonds with its environment. Two nodes are connected by an edge if their respective interaction sites have 'similar' geometry. The length of the edge corresponds to the overall similarity between the two interaction sites; the shorter the edge, the more similar the two binding sites. Nodes that were not part of the main connected component, forming small clusters, have been removed for a clearer view of the network.

Composing the theme dataset for adenine-binding proteins

To generate the dataset of themes for adenine-binding proteins we took the following steps:

Alignments: We used HHSearch (30) to obtain hidden Markov model (HMM) alignments for all the chains in our adenine-binding dataset. We filtered the alignments, keeping only those with E-value under 10^{-2}. For each chain we collected all the alignments to other proteins in the set.

Generate candidate segments for the themes: For each chain, we calculated variations of different minimal lengths: 30, 40, 50, 60, 70, 80 amino acids, and used a unified naming scheme.

Identify the connected components in the chain network: From the alignments generated in the first step we composed a network in which each node is a protein chain, and two nodes are connected by an edge if the respective protein chains are aligned to each other. Next, we separated the chains into connected components. We performed the next step on each connected component separately.

Search and join the variations: For each chain in the adenine-binding dataset, and for each set of variations in the chain, we searched for the connected component of that chain. Each chain was represented by a node in a graph. For each node, we listed all the variations found in it. Starting from a specific node C with a specific variation M_n1, and a specific range of residues (s, e), we considered only edges that connected C via alignments that matched the residues between (s, e). We restricted the edge to connect two nodes with alignment of approximately the same length as (s, e), and for which 80% of the residues of the variation M_n1 were matched to some residues in the alignment.

Theme generation: Once we had the list of pairs of variations that were similar, we grouped them into themes. The themes are the connected components in another graph, where the nodes are the variations we described earlier, and the edges are the similarity relationships between them. We assigned each theme (or connected component) a number. The theme is a set of protein fragments, and evidence of similarity amongst them. The full list of themes can be found in http://trachel-srv.cs.haifa.ac.il/rachel/for_aya/Adenine_related_themes.tar.gz .

Detecting themes in adenine interaction sites

To detect all the themes that bind adenine, we expanded the initial dataset of themes. First, we searched for the themes in the UniProt database (31): we used each theme as an HMM and used HMMER (32), with a threshold of E-value smaller than 10^{-5}. We added the identified matches to the HMM representing each theme and used this new HMM. In order not to lose the proteins that were initially included in the HMM of the theme, we made sure to add them to the resulting HMM. We applied this expansion process twice for each theme. Next, we used HMMER (32) again on our adenine-binding dataset with each theme, and searched for the proteins in this database containing the theme.

Theme network

We listed all the themes found in the interaction site of each of the proteins in our dataset, according to the unified naming scheme described above. We compare all-vs.-all of the proteins in the dataset to search for all the themes shared by pairs of proteins: we created a network where each node represented a protein interaction site, and two nodes were connected by an edge if the corresponding binding sites had a shared theme, hence, amino acids which hydrogen-bonds to adenine in both proteins are part of the same theme (see Figure 5; only clusters with 10 or more nodes are shown). We used Cytoscape (29) and CytoStruct (33) to view the network.

Discover adenine-binding proteins in protein datasets

We used HMMER to search the entire PDB/UniProt databases for each of the themes identified as being shared by the proteins in our dataset of adenine-binding proteins (Figure 5) (34). When a theme was found in a dataset entry with E-value smaller than 10^{-5}, we listed this entry as “suspected adenine binding protein”. When searching against the PDB, we used ComBind to check for adenine-containing ligands in this entry and created two lists. The first list contained proteins that had a theme related to adenine binding, and that also had adenine as part of their structure (possibly in the context of a larger ligand); the second list included proteins that contained a theme related to the binding, but with no adenine in their structure. We used BLAST (35) to search for all the proteins in the second list against proteins from the first list, with the goal of checking whether PDB entries with no bound adenine may share sequence similarity with proteins that do bind adenine. A protein was considered as a probable candidate for binding adenine if it shared at least 80% sequence identity, with 80% coverage, with a protein that was known to bind adenine.

Supplementary Tables and Figures

Reference	Distance (Å)	Angle (°)
Bissants et al. (2010) Journal of Medicinal Chemistry. 53(14): 5061-5084(36)	D-A: 2.8-3.1	130 (D-H…A-A')
McDonald & Thornton (1994) JMB 238: 777-793 (9)	D-A: ≤ 3.9	> 90 (D-H…A-A')
	H-A: ≤ 2.5	
Berndt et al. (1993) JMB 234: 735-750(37)	H-A: ≤ 2.4	≤ 35 (between the proton-donor bond and the line connecting the donor and acceptor)
Baker & Hubbard (1984) Prog. Biophys. Molec. Biol. 44: 97-179 (8)	H-A: ≤ 2.5	+/- 90 ≤ 180 (D-H…A-A')
SwissPDB Viewer (38)	H-A: 1.2 ≤ 2.76	
	D-A: 2.35 ≤ 3.2	
PyMOL (39)	D-A: ≤ 3.6	
RING server (HBexplore program) (40)	D-A: ≤ 3.5	≥ 63 (D-H…A-A')
Arpeggio (7)	≤ radius(H)+radius(A)+0.1Å	≥ 90 (D-H…A=A')

Table S1. Hydrogen-bond definitions, taken from a variety of commonly used tools.

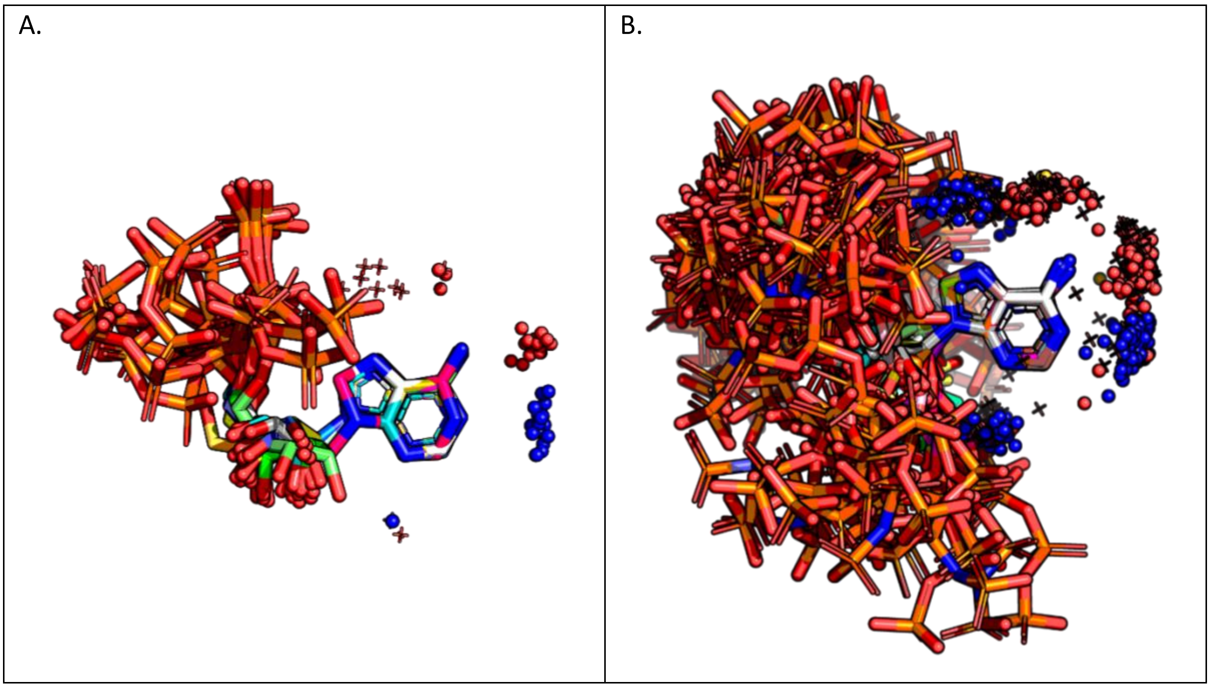

Figure S1. ComBind's results are consistent with the results found by Dennessiouk and co-workers (1-3) when using the same dataset. (*A*) ComBind's results for the original dataset of ATP-binding sites recapitulate Dennessiouk and co-workers' observation that binding is mediated only via the Watson-Crick edge. (*B*) However, ComBind's results for the large dataset of ATP-binding sites show that the original dataset was not diverse enough to detect interactions with all adenine's edges.

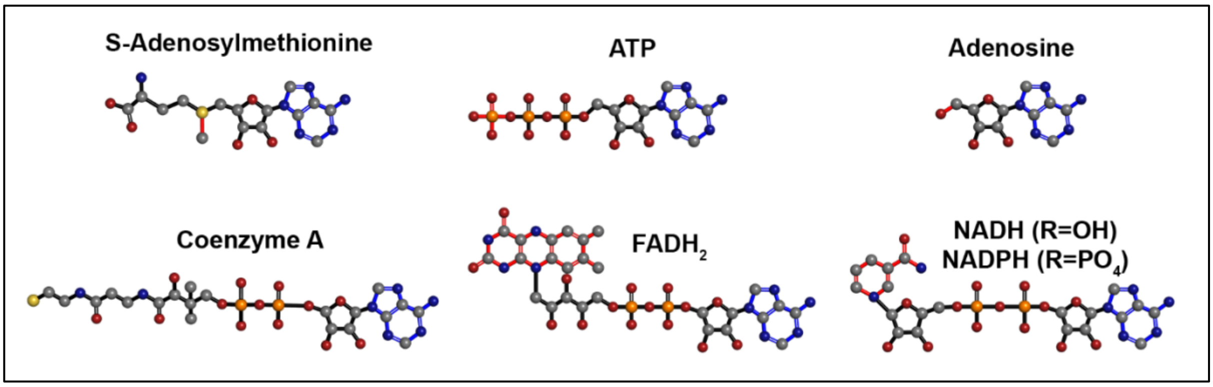

Figure S2. Adenine-containing nucleotide cofactors included in this work. The cofactors are represented as balls and sticks, with carbon atoms marked as grey spheres, nitrogen in blue, oxygen in red, phosphorus in orange and sulfur in yellow. The adenine fragment is marked using blue bonds to separate it from the functional groups, marked using red bonds.

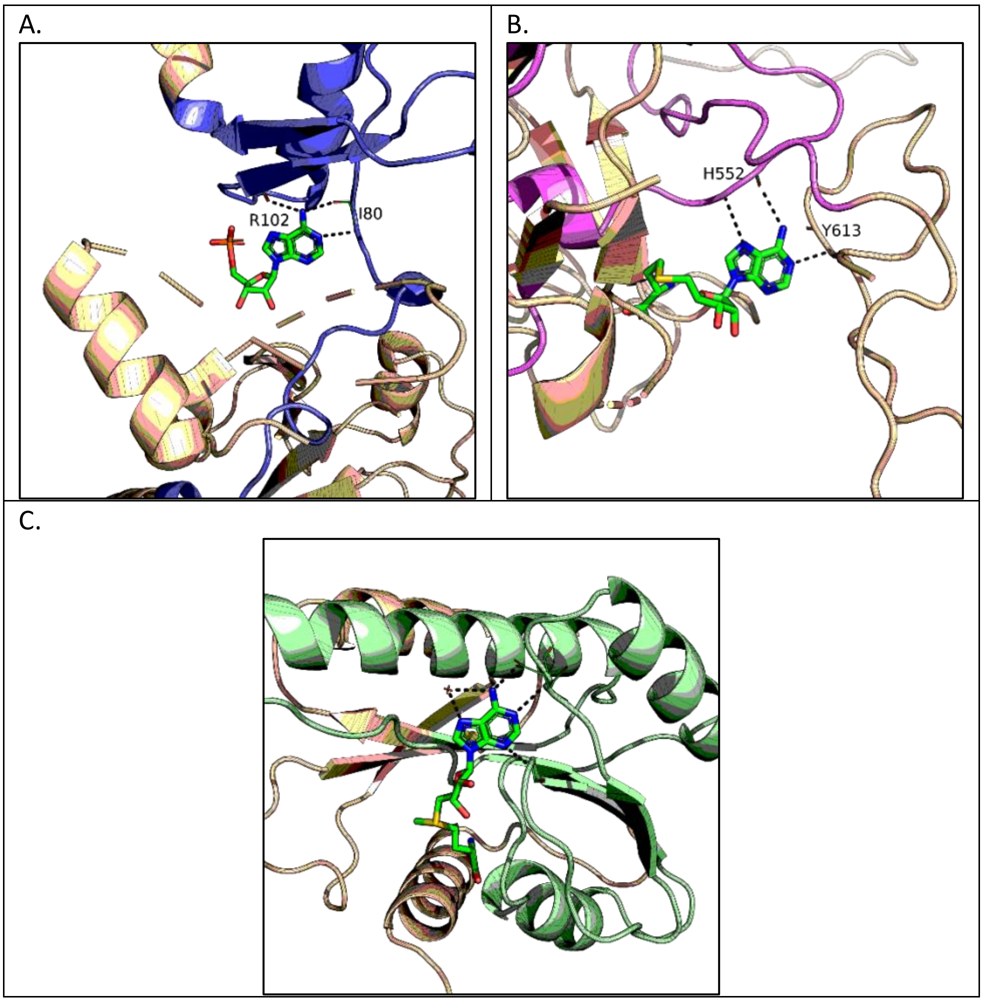

Figure S3. Themes can form adenine-binding patterns in proteins. The protein is shown in wheat with the themes highlighted in colors. The adenine-containing ligand is shown using a bond-stick model, and the hydrogen bonds to specific amino acids of the themes and water molecules are shown in black dashed lines. (*A*) A theme representing the reverse motif with an additional interaction between adenine's N6 in the Hoogsteen edge and a carboxyl group at 'position XV/XVI' (here R102). Demonstrated using PDB 4hg0. (*B*) A theme representing a variation of the reverse motif in the Hoogsteen edge, as found in PDB 4qeo. (*C*) A relatively long theme creating a scaffold for adenine binding, as found in PDB 1ej0. The "Asp" motif is used here, with an additional interaction with adenine's N3. A water molecule forming hydrogen bonds with adenine's Hoogsteen edge is also conserved.

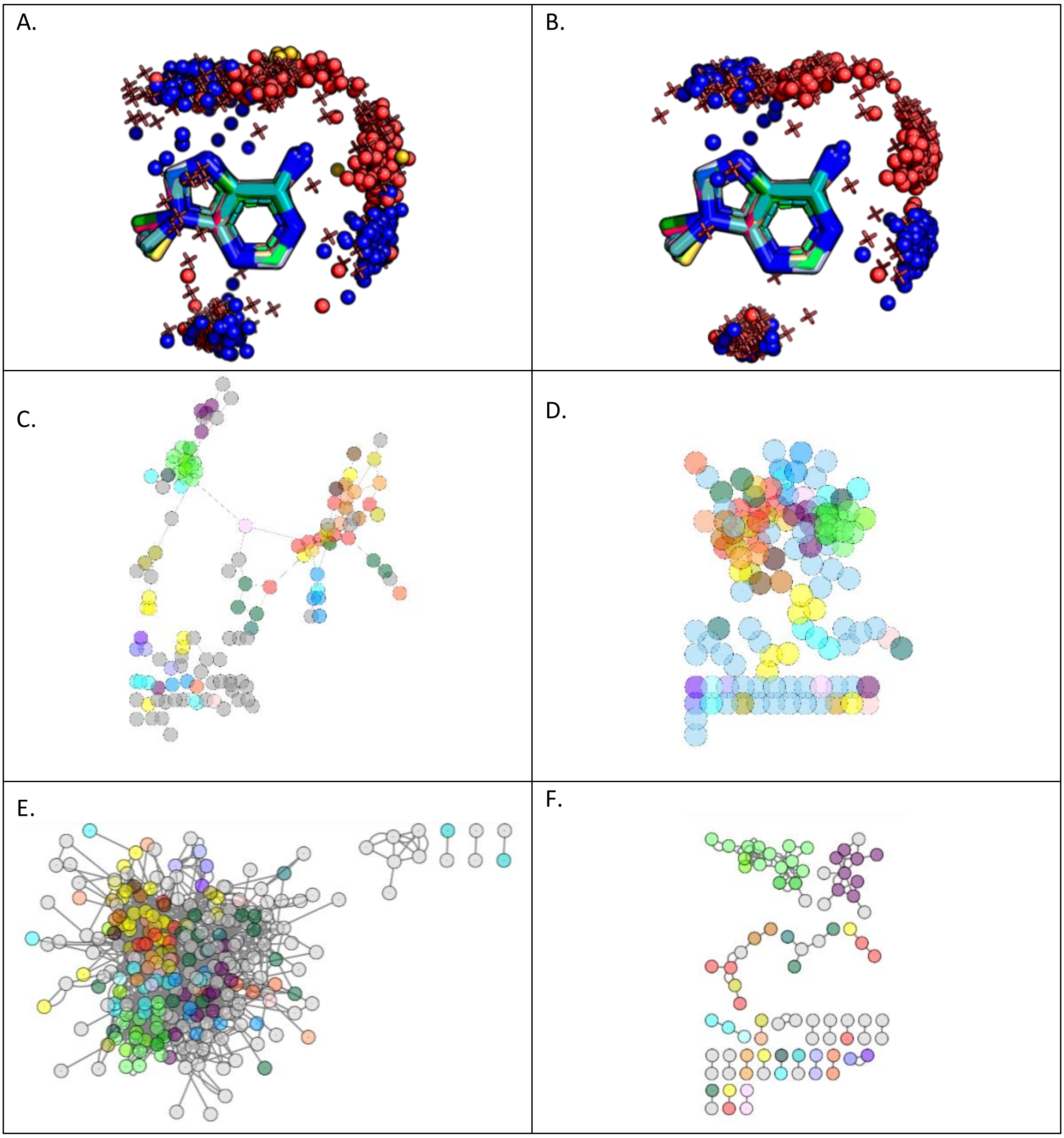

Figure S4. Different hydrogen-bond definitions and geometrical-similarity thresholds may lead to different network representations of protein-ATP complexes, without changing our main conclusions. A network representation of protein-ATP complexes (colored circles) connected based on the geometry of their interaction regions. The nodes are colored according to the PFAM family assignment of the binding protein; only families represented by more than 3 nodes are colored, the rest are in grey. The color scheme is the same as the one used in Figure 4A. (*A*) ComBind's results for the dataset of ATP-binding sites, when the distance threshold for hydrogen bond is 3.9Å. (*B*) Same as *A*, with distance threshold of 3.2Å. (*C*) A network representation of protein-ATP complexes, when the distance threshold for a hydrogen bond is set at 3.9Å. (*D*) Same as *C*, with a threshold of 3.2Å. **E.** Using lax thresholds leads to a larger network, with less noticeable clusters. Two nodes are connected by an edge if the RMSD between the binding sites is under 0.4Å and at least 60% of the interacting atoms are located in close proximity. **F.** Using strict similarity thresholds breaks the network into numerous connected components. Two nodes are connected by an edge if the RMSD between the binding sites is under 0.2Å and at least 70% of the interacting atoms are located in close proximity.

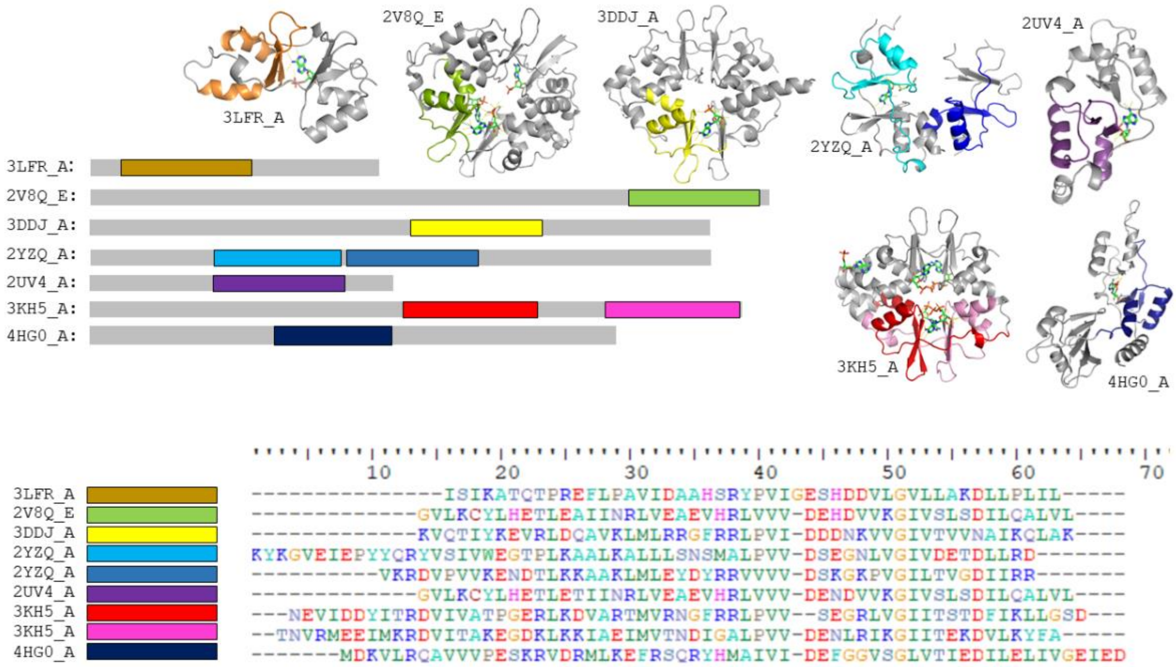

Figure S5. A theme is a set of similar segments that recur, or are 'reused', across protein space: These segments have approximately the same length and their sequences are similar. Here we observe the segments corresponding to an example theme, 'theme 1403', which appears in the adenine-binding sites of proteins in cluster #4 (see supplementary text; "Themes used in adenine binding"). The theme was constructed from nine segments taken from seven chains. We represent each sequence by a cartoon line whose length is proportional to the number of residues in the sequence, and the positions of the segments within them are shown as colored blocks. Similarly, we show the structures of these chains and the colored segments within them. For example, in chain 3LFR_A, there is a brown segment of 48 residues (approximately half of the chain's residues); in chain 2YZQ_A, there are two segments, the first one is colored in cyan, and the second in blue. The bottom of the figure shows the MSA of these segments.

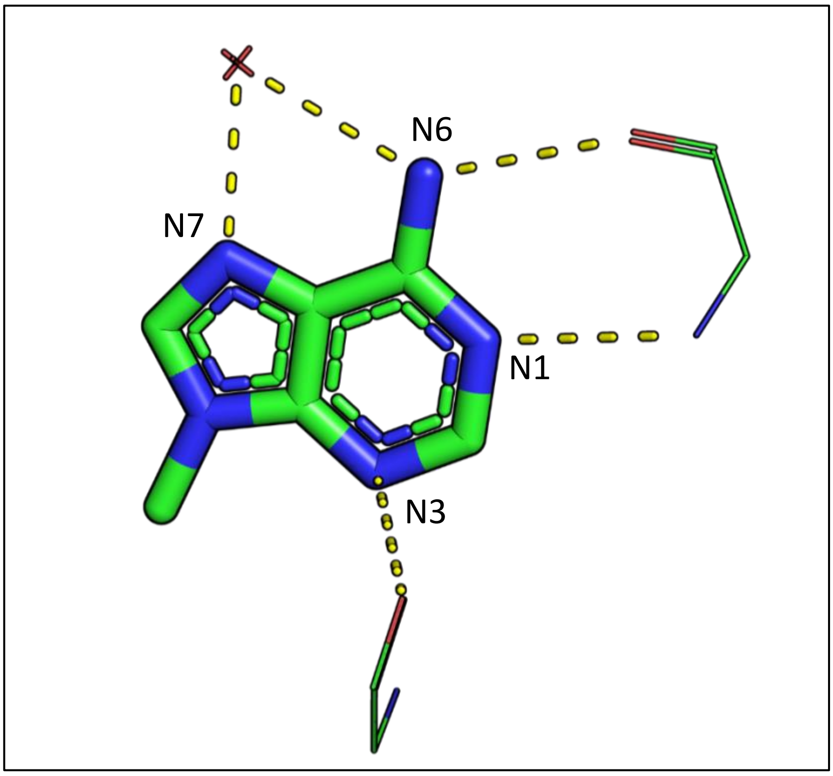

Figure S6. Adenine's binding mode with 1uw1, resembles the mode shared by proteins in cluster 6A in Figure 5. 1uw1 was designed by function (adenine binding) directed in-vitro evolution (41). The protein uses the reverse motif to bind adenine in the Watson-Creek edge, and in addition, a water molecule hydrogen bonds adenine's Hoogsteen edge, while the protein backbone forms another hydrogen bond with adenine's N3.

Dataset S1 (separate file). The list of PDB chains used in the analysis, including their R-free and resolution values, the bound ligand, the atoms that participate in the hydrogen bonds with any of adenine's nitrogen atoms, and ECOD's X- and F-group assignments of the binding atoms.

Dataset S2 (separate file). The list of UniProt accession codes that include one of the themes involved in adenine binding.

PyMOL session for aromatic residues

References

1. K. A. Denessiouk, M. S. Johnson, When fold is not important: a common structural framework for adenine and AMP binding in 12 unrelated protein families. *Proteins* **38**, 310-326 (2000).
2. K. A. Denessiouk, M. S. Johnson, "Acceptor-donor-acceptor" motifs recognize the Watson-Crick, Hoogsteen and Sugar "donor-acceptor-donor" edges of adenine and adenosine-containing ligands. *J Mol Biol* **333**, 1025-1043 (2003).
3. K. A. Denessiouk, V. V. Rantanen, M. S. Johnson, Adenine recognition: a motif present in ATP-, CoA-, NAD-, NADP-, and FAD-dependent proteins. *Proteins* **44**, 282-291 (2001).
4. N. M. O'Boyle *et al.*, Open Babel: An open chemical toolbox. *J Cheminform* **3**, 33 (2011).
5. J. Munkres, Algorithms for the assignment and transportation problems. *Journal of the society for industrial and applied mathematics* **5**, 32-38 (1957).
6. W. Kabsch, A solution for the best rotation to relate two sets of vectors. *Acta Crystallographica Section A: Crystal Physics, Diffraction, Theoretical and General Crystallography* **32**, 922-923 (1976).
7. H. C. Jubb *et al.*, Arpeggio: A Web Server for Calculating and Visualising Interatomic Interactions in Protein Structures. *J Mol Biol* **429**, 365-371 (2017).
8. E. N. Baker, R. E. Hubbard, Hydrogen bonding in globular proteins. *Prog Biophys Mol Biol* **44**, 97-179 (1984).
9. I. K. McDonald, J. M. Thornton, Satisfying hydrogen bonding potential in proteins. *J Mol Biol* **238**, 777-793 (1994).
10. D. F. Stickle, L. G. Presta, K. A. Dill, G. D. Rose, Hydrogen bonding in globular proteins. *Journal of Molecular Biology* **226**, 1143-1159 (1992).
11. P. J. Fleming, G. D. Rose, Do all backbone polar groups in proteins form hydrogen bonds? *Protein Sci* **14**, 1911-1917 (2005).
12. L. Lo Conte, C. Chothia, J. Janin, The atomic structure of protein-protein recognition sites. *J Mol Biol* **285**, 2177-2198 (1999).
13. K. Chen, L. Kurgan, Investigation of atomic level patterns in protein--small ligand interactions. *PloS one* **4**, e4473 (2009).
14. S. K. Panigrahi, G. R. Desiraju, Strong and weak hydrogen bonds in the protein–ligand interface. *Proteins: Structure, Function, and Bioinformatics* **67**, 128-141 (2007).
15. T. Kortemme, A. V. Morozov, D. Baker, An orientation-dependent hydrogen bonding potential improves prediction of specificity and structure for proteins and protein-protein complexes. *Journal of Molecular Biology* **326**, 1239-1259 (2003).

16. J. A. Ippolito, R. S. Alexander, D. W. Christianson, Hydrogen bond stereochemistry in protein structure and function. *J Mol Biol* **215**, 457-471 (1990).
17. P. Chakrabarti, R. Bhattacharyya, Geometry of nonbonded interactions involving planar groups in proteins. *Progress in Biophysics and Molecular Biology* **95**, 83-137 (2007).
18. L. M. Salonen, M. Ellermann, F. Diederich, Aromatic rings in chemical and biological recognition: energetics and structures. *Angew Chem Int Ed Engl* **50**, 4808-4842 (2011).
19. A. Kessel, N. Ben-Tal, *Introduction to proteins: structure, function, and motions* (Taylor & Francis LLC., Boca Raton, Fl., ed. Second, 2018).
20. K. J. Lumb, P. S. Kim, A buried polar interaction imparts structural uniqueness in a designed heterodimeric coiled coil. *Biochemistry* **34**, 8642-8648 (1995).
21. D. N. Bolon, S. L. Mayo, Polar residues in the protein core of Escherichia coli thioredoxin are important for fold specificity. *Biochemistry* **40**, 10047-10053 (2001).
22. D. Petrey, B. Honig, Free energy determinants of tertiary structure and the evaluation of protein models. *Protein Sci* **9**, 2181-2191 (2000).
23. C. M. Baker, G. H. Grant, Role of aromatic amino acids in protein–nucleic acid recognition. *Biopolymers* **85**, 456-470 (2007).
24. J. L. Asensio, A. Arda, F. J. Canada, J. Jimenez-Barbero, Carbohydrate-aromatic interactions. *Acc Chem Res* **46**, 946-954 (2013).
25. D. R. Ripoll, C. H. Faerman, P. H. Axelsen, I. Silman, J. L. Sussman, An electrostatic mechanism for substrate guidance down the aromatic gorge of acetylcholinesterase. *Proceedings of the National Academy of Sciences of the United States of America* **90**, 5128-5132 (1993).
26. M. Cammisa, A. Correra, G. Andreotti, M. V. Cubellis, Identification and analysis of conserved pockets on protein surfaces. *BMC bioinformatics* **14 Suppl 7**, S9-S9 (2013).
27. M. Steinegger, J. Soding, MMseqs2 enables sensitive protein sequence searching for the analysis of massive data sets. *Nat Biotechnol* **35**, 1026-1028 (2017).
28. M. Steinegger, J. Soding, Clustering huge protein sequence sets in linear time. *Nat Commun* **9** (2018).
29. R. Saito *et al.*, A travel guide to Cytoscape plugins. *Nat Methods* **9**, 1069-1076 (2012).
30. J. Soding, Protein homology detection by HMM-HMM comparison. *Bioinformatics* **21**, 951-960 (2005).
31. T. UniProt Consortium, UniProt: the universal protein knowledgebase. *Nucleic Acids Res* **46**, 2699 (2018).
32. S. R. Eddy, Accelerated Profile HMM Searches. *PLoS Comput Biol* **7**, e1002195 (2011).
33. S. Nepomnyachiy, N. Ben-Tal, R. Kolodny, CyToStruct: Augmenting the Network Visualization of Cytoscape with the Power of Molecular Viewers. *Structure* **23**, 941-948 (2015).
34. E. Boutet *et al.*, UniProtKB/Swiss-Prot, the Manually Annotated Section of the UniProt KnowledgeBase: How to Use the Entry View. *Methods Mol Biol* **1374**, 23-54 (2016).
35. S. F. Altschul, W. Gish, W. Miller, E. W. Myers, D. J. Lipman, Basic local alignment search tool. *J Mol Biol* **215**, 403-410 (1990).
36. C. Bissantz, B. Kuhn, M. Stahl, A medicinal chemist's guide to molecular interactions. *J Med Chem* **53**, 5061-5084 (2010).

37. K. D. Berndt, P. Guntert, K. Wuthrich, Nuclear magnetic resonance solution structure of dendrotoxin K from the venom of Dendroaspis polylepis polylepis. *J Mol Biol* **234**, 735-750 (1993).
38. N. Guex, M. C. Peitsch, SWISS-MODEL and the Swiss-PdbViewer: an environment for comparative protein modeling. *Electrophoresis* **18**, 2714-2723 (1997).
39. Anonymous (2017) The PyMOL Molecular Graphics System, Version 2.0.
40. D. Piovesan, G. Minervini, S. C. E. Tosatto, The RING 2.0 web server for high quality residue interaction networks. *Nucleic Acids Res* **44**, W367-W374 (2016).
41. P. L. Surdo, M. A. Walsh, M. Sollazzo, A novel ADP-and zinc-binding fold from function-directed in vitro evolution. *Nature structural & molecular biology* **11**, 382 (2004).
